# Management von Kardiomyopathien: Neue ESC-Leitlinie 2023

**DOI:** 10.1007/s00059-023-05224-z

**Published:** 2023-12-05

**Authors:** Noemi Pavo, Christian Hengstenberg

**Affiliations:** Klinische Abteilung für Kardiologie, Universitätsklinik für Innere Medizin II, Medizinische Universität Wien, Währinger Gürtel 18–20, 1090 Wien, Österreich

**Keywords:** Herzmuskelerkrankungen, Phänotypen, Diagnostik, Management, Empfehlungen, Heart muscle diseases, Phenotypes, Diagnostics, Management, Recommendations

## Abstract

Die Gruppe der Kardiomyopathien hat in den letzten Jahren verstärkt Aufmerksamkeit erhalten, nachdem einige ihrer Ursachen identifiziert und sie mithilfe moderner Bildgebungsmethoden genauer charakterisiert werden konnten. Regelmäßig wurden von nationalen und internationalen Fachgesellschaften neue Definitionen und Klassifikationsschemata bereitgestellt. Die neue Leitlinie der European Society of Cardiology (ESC) von 2023 zum Management der Kardiomyopathien ist nun international die erste Guideline, die umfassend alle Kardiomyopathien in einem Dokument behandelt. Es handelt sich um eine neue Leitlinie, sodass die meisten Empfehlungen ebenso neu sind. Eine Ausnahme bildet der Abschnitt zur hypertrophen Kardiomyopathie (HCM), bei dem es sich um eine Aktualisierung der ESC-Leitlinie von 2014 zur Diagnose und Behandlung der HCM handelt. Das Hauptziel dieser Leitlinie besteht darin, einen klaren Leitfaden für die Diagnose von Kardiomyopathien bereitzustellen, allgemeine Bewertungs- und Managementprobleme zu betonen und den Leser auf die relevante wissenschaftliche Evidenzbasis für die Empfehlungen hinzuweisen. Aufgrund des Umfangs können keine detaillierten Beschreibungen und Empfehlungen für jede spezifische Kardiomyopathie bereitgestellt werden, jedoch wird auf die entsprechende Literatur verwiesen.

## Abstract (en)

The group of cardiomyopathies has received increasing attention over the last few years after some of the causes were identified and they could be characterized more exactly using modern imaging methods. New definitions and classification schemes were regularly provided by national and international cardiac societies. The new guidelines of the European Society of Cardiology (ESC) from 2023 on the management of cardiomyopathies are the first guidelines that comprehensively address all cardiomyopathies in one document. As these are new guidelines most of the recommendations are also new. An exception is the section on hypertrophic cardiomyopathy (HCM), which provides a targeted update of the 2014 ESC guidelines on the diagnosis and treatment of HCM. The main aim of the guidelines is to provide clear guidance for the diagnosis of cardiomyopathies, to highlight general assessment and management problems and to point out the relevant scientific evidence for the recommendations to the readership. Due to the magnitude detailed descriptions and recommendations cannot be provided for each individual cardiomyopathy phenotype; however, reference is made to the relevant literature.

Die ESC(European Society of Cardiology)-Leitlinie von 2023 für das Management der Kardiomyopathien (CMP) stellt international die erste Leitlinie dar, die alle CMP gemeinsam behandelt [1]. Da es sich um eine neue Leitlinie handelt, sind die meisten Empfehlungen ebenfalls neu. Aufgrund der begrenzten Anzahl randomisierter, kontrollierter Studien (RCT) auf diesem Gebiet haben die meisten Empfehlungen den Evidenzgrad C, entsprechend dem Expertenkonsens der Task Force (Abb. 1). Eine Ausnahme bilden die hypertrophen CMP (HCM), für die bereits eine separate ESC-Leitlinie von 2014 existiert [2]. Diese wurde nun in die neue Leitlinie integriert, und die Empfehlungen wurden gemäß dem aktuellen Kenntnisstand überarbeitet. Das Hauptziel dieser Leitlinie besteht darin, den diagnostischen Ansatz sowie das allgemeine Management von CMP zu beschreiben. Es sei jedoch darauf hingewiesen, dass sie aufgrund des Umfangs keine detaillierte Beschreibung und Empfehlungen für die einzelnen spezifischen Ursachen von CMP enthält. Auch wird keine Verknüpfung zu den bereits bestehenden Leitlinien der Perikarderkrankungen hergestellt [3, 4]. Insgesamt stellt diese Leitlinie eine sinnvolle Ergänzung zu den bestehenden Leitlinien dar und betont die Bedeutung der Ursachenforschung bei Herzmuskelerkrankungen.

**Figure 1:**
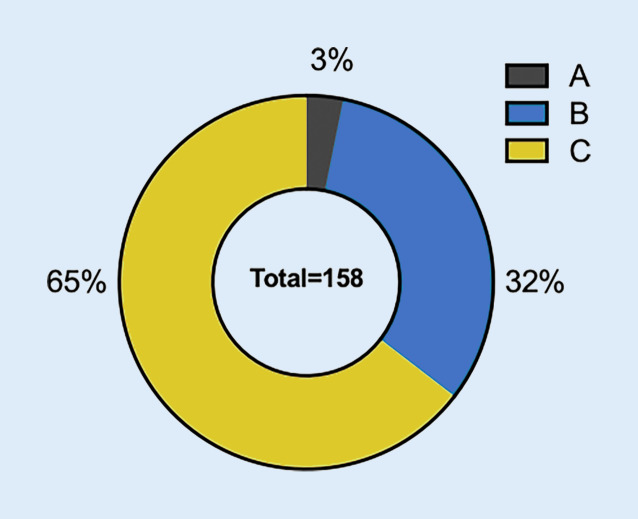

Die Struktur und die wesentlichen Punkte der neuen Leitlinie sind in Abb. 2 dargestellt. Die wichtigsten Innovationen der CMP-Leitlinie sind:

- phänotypbasierter diagnostischer Pfad mit Charakterisierung eines neuen Phänotyps, der nichtdilatativen linksventrikulären CMP (NDLVC);
- Unterstreichen der Rolle der kardialen Magnetresonanztomographie (cMRT) zur Feststellung von Narben;
- Betrachten der CMP als eine mögliche Grundlage verschiedener klinischer Präsentationen wie Herzinsuffizienz oder Arrhythmie;
- überarbeitete Empfehlungen zum genetischen Testen von Indexpatienten und Angehörigen;
- Berücksichtigung des Patientenwegs von der Kindheit bis ins Erwachsenenalter;
- neue Empfehlungen bezüglich Risikoabschätzung von plötzlichem Herztod („sudden cardiac death“, SCD) in den verschiedenen Phänotypen;
- Update zum Management der linksventrikulären Obstruktion bei hypertropher CMP (HCM).

**Figure 2:**
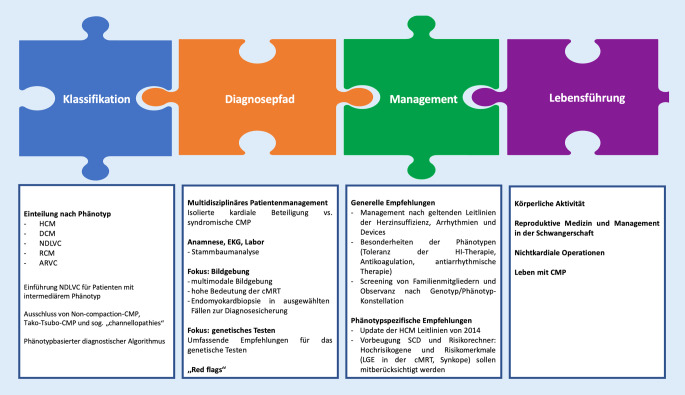

## Was sind Kardiomyopathien, und wieso ist es wichtig, sie zu definieren?

Der Begriff „CMP“ beschreibt allgemein eine Erkrankung des Herzmuskels, die sich durch strukturelle oder funktionelle Abnormitäten auszeichnet [5]. Historisch wurde eine CMP, die durch eine Ischämie, also zumeist durch eine koronare Herzkrankheit (KHK), verursacht wurde, von der engeren Bezeichnung „CMP“ ausgeschlossen. Zur Kontrastierung wurde auch der Begriff „nichtischämische CMP“ eingeführt. Im Laufe der Jahre erhielten die CMP zunehmend Aufmerksamkeit, nachdem einige ihrer Ursachen identifiziert und sie mithilfe moderner Bildgebungsverfahren besser charakterisiert werden konnten. Es wurden regelmäßig neue Definitionen und Klassifikationsschemata von der World Health Organization (WHO) sowie von nationalen und internationalen Fachgesellschaften bereitgestellt.

Die aktuelle Leitlinie konzentriert sich auf CMP, die nicht ausreichend durch KHK, Hypertonie, valvuläre Herzerkrankungen oder angeborene Herzfehler erklärt werden können. Es ist jedoch möglich, dass mehrere Ursachen gleichzeitig vorliegen. CMP können klinisch unauffällig sein und nur zufällig entdeckt werden oder sich aber primär in einer Arrhythmie oder einer Herzinsuffizienz manifestieren. Die Diagnose einer CMP sollte unbedingt die Suche nach ihrer Ursache zur Folge haben. Das Wissen über die Ursache hat Auswirkungen auf die Patientenaufklärung, die Prognoseabschätzung, die Bewertung des Risikos (insbesondere hinsichtlich des Risikos für SCD), das generelle Management und in einigen speziellen Fällen auch auf die Therapie. Bei genetischen CMP spielt auch die Abschätzung des Risikos, die Indikationsstellung zum genetischen Testen, die Früherkennung oder die Therapie der betroffenen Familienmitglieder eine wichtige Rolle. Zusätzlich sollte eine mögliche Weitergabe an Nachkommen bei Familienplanungsentscheidungen bedacht werden.

## Phänotypen der Kardiomyopathien

Schon im Jahr 1961 schlug Goodwin eine Klassifizierung der CMP, basierend auf ihrem Phänotyp, vor [6]. Obwohl damals diskutiert wurde, dass diese rein deskriptive Einteilung, die aufgrund des begrenzten Wissens über die Ursachen der meisten Herzmuskelerkrankungen eingeführt wurde, wahrscheinlich vorübergehend und nur eingeschränkt anwendbar sei, blieb diese morphologische Klassifikation bis heute größtenteils unverändert. Im Jahr 2008 schlug das Konsensdokument der ESC einen pragmatischen Ansatz vor, nämlich CMP neben ihrem Phänotyp auch nach familiärer Häufung zu klassifizieren. Die 4 Phänotypen HCM, dilatative (DCM), arrhythmogene rechtsventrikuläre (ARVC) und restriktive CMP (RCM) sowie eine separate Kategorie für nichtklassifizierte CMP sollten in familiäre/genetische und nichtfamiliäre/nichtgenetische Formen unterteilt werden [3]. Die aus dem amerikanischen Raum stammende MOGES-Klassifikation aus dem Jahre 2014 versucht, die CMP noch ganzheitlicher zu erfassen, indem sie neben Phänotyp (M = „morphology“) und genetischer Beteiligung (G = „genetic inheritance pattern“) auch die Beteiligung anderer Organsysteme (O = „organ involvement“) sowie Status (S = „stage“) und Ätiologie (E = „etiology“) zuordnet [7]. Die aktuelle Leitlinie hat nun die 4 Phänotypen übernommen und um einen neuen Phänotyp, nämlich NDLVC, erweitert. Die genetische Grundlage erhält in der Leitlinie einen großen Stellenwert. Im Gegensatz dazu werden jedoch die Inflammation als Ätiologie und die Gruppe der inflammatorischen CMP nicht gesondert hervorgehoben. Eine detaillierte Beschreibung der Phänotypen findet sich in Tab. 1.

**Table 1:** 

Phänotyp	Abkürzung	Definition
Hypertrophe CMP	HCM	Erhöhte linksventrikuläre Wanddicke (mit oder ohne rechtsventrikuläre Hypertrophie) oder Masse, die nicht allein durch abnormale Füllungsdrücke erklärt werden kann
Dilatative CMP	DCM	Dilatation des linken Ventrikels mit einer globalen oder regionalen systolischen Dysfunktion, die nicht allein durch abnormale Füllungsdrücke erklärt werden kann
Nichtdilatative linksventrikuläre CMP	NDLVC	Vorhandensein von linksventrikulären nichtischämischen Narben oder Fetteinlagerungen, unabhängig vom Vorhandensein globaler oder regionaler Wandbewegungsstörungen, oder eine isolierte globale linksventrikuläre Hypokinesie ohne Narbenbildung
Arrhythmogene rechtsventrikuläre CMP	ARVC	Überwiegend rechtsventrikuläre Dilatation und/oder Dysfunktion bei gleichzeitiger histologischer Beteiligung und/oder elektrokardiographischen Abnormalitäten
Restriktive CMP	RCM	Restriktive links- und/oder rechtsventrikuläre Pathophysiologie bei gleichzeitig normalen oder reduzierten diastolischen Volumina (von einem oder beiden Ventrikeln), normalen oder reduzierten systolischen Volumina und normaler Wanddicke der Ventrikel

Die NDLVC umfasst Patienten mit einer isolierten Dysfunktion des linken Ventrikels ohne Anzeichen von Narbenbildung oder Patienten mit nichtischämischer Narbenbildung, unabhängig vom Vorliegen einer systolischen Dysfunktion. Dadurch können nun auch Patienten erfasst werden, bei denen trotz des Vorliegens einer Myokarderkrankung keine der Definitionen der anderen Klassen zutrifft (sog. intermediäre Phänotypen).

## Integriertes Patientenmanagement

Die Betreuung von CMP erfordert einen koordinierten, systematischen und individuell angepassten Ansatz mit optimaler Versorgung durch ein multidisziplinäres Expertenteam. Die Zusammensetzung dieses Teams richtet sich nach den spezifischen Bedürfnissen des Patienten und seiner Familie. Ein besonderes Augenmerk liegt dabei auf der nahtlosen Fortführung der Betreuung von Jugendlichen beim Erreichen des Erwachsenenalters. Die Leitlinie betont, dass alle Patienten und deren Angehörige Zugang zu solchen multidisziplinären Teams haben sollten (I C). Ebenso sollte eine rechtzeitige und angemessene Vorbereitung auf den Übergang von der pädiatrischen zur Erwachsenenversorgung für Jugendliche mit CMP erfolgen (I C).

## Phänotyporientierter diagnostischer Pfad

Die Identifikation eines CMP-Phänotyps markiert lediglich den Beginn eines diagnostischen Pfades, dessen Hauptziel die Ermittlung der zugrunde liegenden Ursache ist [8]. Die Gründe für die Erstvorstellung von Patienten mit CMP sind vielfältig und decken ein weites Spektrum ab. Patienten können aufgrund kardialer Symptome, elektrokardiographischer Auffälligkeiten, Herzrhythmusstörungen, SCD, manifester Herzinsuffizienz, auffälliger Ergebnisse beim Familienscreening oder aufgrund von Multiorganerkrankungen zur Abklärung kommen. Patienten mit einer CMP weisen auch möglicherweise nur geringfügige oder gar keine kardialen Symptome auf. Umgekehrt können nichtkardiale Symptome wichtige Hinweise auf die zugrunde liegende Ursache sein.

Wenn der Verdacht auf eine CMP besteht oder eine CMP bestätigt ist, wird eine multiparametrische Herangehensweise mit klinischer Untersuchung, Stammbaumanalyse, Elektrokardiogramm (EKG), Holter-Monitoring, Labortests und multimodaler Bildgebung empfohlen (I C). Die Stammbaumanalyse sollte mindestens 3 bis 4 Generationen umfassen, um bei der Diagnosestellung zu helfen, den Vererbungsmodus zu bestimmen, Hinweise auf den Krankheitsverlauf zu liefern und Familienmitglieder mit erhöhtem Risiko zu identifizieren (I C). Eine negative Stammbaumanalyse schließt jedoch das Vorhandensein einer genetischen CMP nicht aus. Obwohl einige Veränderungen im EKG eher unspezifisch sind, wie z. B. ein Atrioventrikular(AV)-Block, Präexzitation oder niedrige QRS-Voltage, können sie dennoch Hinweise auf die Ursache der CMP liefern. In Bezug auf Laboruntersuchungen unterscheidet die Leitlinie zwischen First-level- (I C) und Second-level-Tests (IIa C).

### Multimodale Bildgebung

Multimodale Bildgebung ist ein zentrales Element des diagnostischen Work-up. Die Empfehlungen hierzu sind in Tab. 2 zusammengefasst. Die Leitlinie betont die Bedeutung von standardisierten Protokollen sowie den bidirektionalen Informationsaustausch zwischen dem überweisenden Arzt und dem bildgebenden Spezialisten, um die neuen Ergebnisse korrekt interpretieren zu können.

**Table 2:** 

	Empfehlungsgrad	Evidenz
*Echokardiographie*		
Eine umfassende Bewertung der Herzdimensionen von LV und RV systolisch (global und regional) sowie der diastolischen LV-Funktion wird für alle Patienten mit CMP initial sowie im Verlauf empfohlen, um den Krankheitsverlauf zu überwachen sowie Risikostratifizierung und Management zu unterstützen	I	B
*Kardiale Magnetresonanztomographie (cMRT)*		
KM-unterstützte cMRT wird für alle Patienten mit CMP bei initialer Präsentation empfohlen	I	B
KM-unterstützte cMRT sollte für das Follow-up erwogen werden, um den Krankheitsverlauf zu überwachen sowie Risikostratifizierung und Management zu unterstützen	IIa	C
KM-unterstützte cMRT sollte für die Evaluierung der Therapieantwort bei Amyloidose, M. Fabry, Sarkoidose und anderen inflammatorischen CMP sowie Hämochromatose bei kardialer Beteiligung erwogen werden	IIa	C
KM-unterstützte cMRT sollte in Familien mit CMP, bei denen eine krankheitsverursachende Variante identifiziert wurde, für die Diagnose und Früherkennung bei genotyppositiven/phänotypnegativen Familienmitgliedern in Betracht gezogen werden	IIa	B
KM-unterstützte cMRT kann in Familien mit CMP, bei denen keine genetische Diagnostik vorliegt, für die Diagnose und die Früherkennung bei phänotypnegativen Familienmitgliedern in Betracht gezogen werden	IIb	C
*Kardiale Computertomographie (cCT)*		
KM-unterstützte cCT sollte bei Patienten mit Verdacht auf CMP und unzureichender Bildqualität in der Echokardiographie oder bei Kontraindikation für eine cMRT in Betracht gezogen werden	IIa	C
KM-unterstützte cCT sollte bei Patienten mit Verdacht auf CMP in Betracht gezogen werden, um eine angeborene oder erworbene Erkrankung der Koronarien als Ursache der CMP auszuschließen	IIa	C
*Nuklearmedizinische Untersuchungen*		
^18^F‑FDG-PET sollte bei Patienten mit Verdacht auf eine Sarkoidose zur diagnostischen Abklärung in Betracht gezogen werden	IIa	C
DPD/PYP/HMDP-Knochenszintigraphie wird bei Patienten mit Verdacht auf kardiale ATTR-Amyloidose zur Diagnosefindung empfohlen	I	B
*Endomyokardbiopsie (EMB)*		
EMB wird bei Patienten empfohlen, bei denen die klinischen Untersuchungen auf eine entzündliche, infiltrative oder eine Speichererkrankung hindeuten und die Diagnose mit anderen Mitteln nicht sichergestellt werden kann [9]	IIa	C

*LV* linker Ventrikel, *RV* rechter Ventrikel, *CMP* Kardiomyopathie, *KM* Kontrastmittel, *FDG* Fluordesoxyglukose, *PET* Positronenemissionstomographie, *DPD/PYP/HMDP* „3,3-diphosphono‑1,2‑propanodicarboxylic acid“/„pyrophosphate“/„hydroxymethylene diphosphonate“, *ATTR* Transthyretinamyloidose

### Genetisches Testen

CMP sind durch eine bemerkenswerte genetische und allelische Vielfalt gekennzeichnet. Das bedeutet, dass viele verschiedene Varianten in zahlreichen Genen den gleichen Phänotyp auslösen können. Darüber hinaus tritt die Erkrankung nicht bei allen Personen mit einer verursachenden genetischen Mutation auf, und bei denjenigen, die eine CMP entwickeln, gibt es eine erhebliche Bandbreite in Bezug auf das Alter und den Schweregrad der Manifestation [10, 11]. Diese Variabilität könnte auf einer genetischen Heterogenität der Variante, auf dem zusätzlichen Beitrag nichtgenetischer Faktoren, aber auch auf anderen genetischen Faktoren, die die ursprüngliche Mendel-Vererbung verstärken oder abschwächen, beruhen.

Genomweite Assoziationsstudien (GWAS) deuten darauf hin, dass sowohl bei der DCM als auch bei der HCM ein polygener Hintergrund mit komplexer Vererbung häufig vorkommt. Im Gegensatz zu einer seltenen Mutation mit großem Effekt liegt hier eher eine Co-Vererbung mehrerer Suszeptibilitätsgene mit variablem Effekt vor. Insgesamt reicht die Bandbreite von der klassischen Mendel-Vererbung bis hin zu polygenen CMP mit vielen Varianten und teilweise unbekanntem Effekt. Das Testen in Familien, bei denen eine monogene CMP unwahrscheinlich ist, repräsentiert eine relativ neue Anwendung der Methode, die zwar Evidenz generiert, aber noch keine wirklichen Implikationen hat. In Zukunft könnten polygene Risikoscores die kumulative Auswirkung vieler Varianten mit kleinem Effekt besser abbilden und das Management steuern.

Genetisches Testen von Patienten mit CMP, die einem Mendel-Erbmuster folgen, ist mittlerweile zum klinischen Standard im Management dieser Patienten geworden. Das genetische Testen sollte dann durchgeführt werden, wenn es für das betroffene Individuum von Vorteil ist, um die Diagnose zu bestätigen, die Prognose abzuschätzen, die Therapie anzupassen und die Familienplanung zu informieren. Darüber hinaus kann das Testen auch angezeigt sein, wenn es zwar nicht unbedingt für das betroffene Individuum selbst, aber für Verwandte von Nutzen ist, z. B. weil sie sonst in ein langjähriges Beobachtungsprogramm aufgrund des Verdachts einer familiären CMP eingeschlossen würden. Die Leitlinie enthält eine Tabelle, in der alle Gene aufgeführt sind, deren Varianten mit monogenen, nichtsyndromischen CMP in Verbindung gebracht wurden. Der Beitrag dieser Gene zur Manifestation der jeweiligen CMP ist farbkodiert. Bei verschiedenen Formen der CMP, die früher aufgrund externer Faktoren als sekundär angesehen wurden, wurde kürzlich eine genetische Rolle nachgewiesen, was als Second-hit-Theorie bezeichnet wird. Die Möglichkeit einer genetischen Prädisposition sollte bei der Erfassung der Familienanamnese und bei der Entscheidung über genetisches Testen in Betracht gezogen werden. Die genauen Empfehlungen sind in Tab. 3 aufgelistet.

**Table 3:** 

	Empfehlungsgrad	Evidenz
*Genetische Beratung*		
Ein genetisches Beratungsgespräch, durchgeführt von einem „healthcare professional“ mit Expertise in Humangenetik, wird für Familien mit genetischen CMP oder V. a. genetische CMP empfohlen zur Entscheidungsfindung und zur psychischen Unterstützung	I	B
Genetisches Testen sollte mit Hintergrund eines multidisziplinären Teams durchgeführt werden, das eine entsprechende Expertise bezüglich der Methode, der Interpretation der Sequenzvarianten und der klinischen Anwendung der Erkenntnisse hat, typischerweise in einem Zentrum für CMP oder einem Netzwerk mit Zugriff auf gleichwertiges Fachwissen	I	B
Prä- und Posttestberatung wird für alle Individuen, die einem genetischen Testen unterzogen werden, empfohlen	I	B
Falls eine Pränataldiagnostik seitens der Familie durchgeführt wird, sollte dies zu einem frühen Zeitpunkt der Schwangerschaft geschehen, um die Entscheidungsfindung bezüglich Fortführung oder Koordination der Schwangerschaft zu ermöglichen	I	C
Ein Gespräch über genetisches Testen in der reproduktiven Medizin, durchgeführt von „healthcare professionals“ mit Expertise in Humangenetik, sollte allen Familien mit genetischen CMP angeboten werden	IIa	C
*Indexfall – Patient*		
Genetisches Testen wird bei Patienten empfohlen, die die Diagnosekriterien für eine CMP erfüllen und bei denen es eine Diagnose, eine Prognose und eine therapeutische Einordnung oder ein reproduktives Management der Patienten ermöglicht, oder in Fällen, bei denen es eine Kaskadengenetik zur Evaluierung der Familienangehörigen zur Folge haben könnte, die sonst in Beobachtungsprogramme eingeschlossen werden müssten	I	B
Genetisches Testen wird für Individuen mit einer CMP, die *post mortem* festgestellt wurde, empfohlen, sofern eine Diagnose die Behandlung der überlebenden Angehörigen vereinfacht	I	C
Genetisches Testen kann bei Patienten, die die Diagnosekriterien für eine CMP erfüllen, in Betracht gezogen werden, falls es einen Nettonutzen für den Patienten einschließlich psychologischer Faktoren und Patentenwunsch bringt, auch dann, wenn es keine Implikationen bezüglich Diagnose, Prognose, Therapie und Testen der Angehörigen hat	IIb	C
Genetisches Testen kann bei Patienten mit einer Borderline-CMP, die die Diagnosekriterien für eine CMP nicht gänzlich erfüllen, nach Evaluierung durch ein Spezialistenteam erwogen werden	IIb	C
*Angehörige*		
Kaskadentesten mit Prä- und Posttestberatung wird für Erwachsene mit Risiko empfohlen, falls eine genetische Diagnose (P/LP-Variante) einer CMP bei einem Familienangehörigen gesichert wurde (beginnend bei den Verwandten 1. Grades und mit sequenzieller Fortführung)	I	B
Kaskadentesten mit Prä- und Posttestberatung wird für Kinder mit Risiko empfohlen, falls eine genetische Diagnose (P/LP-Variante) einer CMP bei einem Familienangehörigen gesichert wurde (beginnend bei den Verwandten 1. Grades und mit sequenzieller Fortführung), wobei die Art der CMP, das Alter, der Onset und die Präsentation in der Familie sowie klinische und legale Aspekte berücksichtigt werden sollen	IIa	B
Das Testen einer „variant of unknown significance“ (VUS), typischerweise bei den Eltern oder bei betroffenen Verwandten, sollte in Erwägung gezogen werden, um die Segregation der Variante und der CMP in der Familie zu bestimmen und damit die Relevanz der Variante festzustellen	IIa	C
Genetisches Testen sollte nicht bei phänotypnegativen Verwandten von Patienten mit einer CMP, bei denen kein sicherer genetischer Hintergrund (P/LP-Variante) festgestellt wurde, durchgeführt werden	III	C

*P/LP* „pathogenic/likely pathogenic“, *CMP* Kardiomyopathie

Es wurde immer wieder versucht, die ursächlichen Gene nach dem assoziierten klinischen Phänotyp zu gruppieren. So gilt beispielsweise die HCM als Erkrankung des Sarkomers und die ARVC als Erkrankung der Desmosomen. Jedoch hat sich mit der Zeit herausgestellt, dass die Genotyp-Phänotyp-Korrelation komplex ist, wie es im Folgenden anhand von Mutationen im *MYH7*(„myosin heavy chain 7“)- und im *ACTC1*(„actin alpha cardiac muscle 1“)- sowie im *DES*(Desmin)-Gen dargestellt ist.

*MYH7* kodiert für die schwere Kette des Beta-Myosins, eines Proteins des Sarkomers. Bereits in den frühen 1990er-Jahren wurden Mutationen im *MYH7*-Gen als eine Ursache für HCM identifiziert. Mutationen in *MYH7* und *MYBPC3* („cardiac myosin binding protein C“) sind zusammen für etwa die Hälfte aller Fälle einer genetischen HCM verantwortlich [12]. Später wurden *MYH7*-Mutationen auch bei der DCM beschrieben. Hier sind sie für 1–5 % kasual verantwortlich und damit die dritthäufigste Ursache für die genetische DCM [13]. In einer großen Kohorte von Patienten mit DCM mit *MYH7*-Mutationen zeigte sich, dass die Erkrankung durch eine hohe altersabhängige phänotypische Expression charakterisiert ist [14]. Die Herzinsuffizienz steht meist im Vordergrund, arrhythmische Events sind im Vergleich zur LMNA(Lamin A/C)-CMP wesentlich seltener [14]. *MYH7*-Varianten wurden auch bei CMP mit „non-compaction“ und Ebstein-Anomalie sowie bei der NDLVC und der RCM gefunden.

*ACTC1* kodiert für „α-cardiac actin“ und ist eine der Hauptkomponenten der dünnen Filamente, die zusammen mit Troponin und Tropomyosin mit dem MYH7-Protein interagieren, um Kraft für die Muskelkontraktion zu generieren. Mutationen in *ACTC1* sind eine seltene Ursache für HCM, jedoch neigen pathogene Varianten dazu, einen großen Effekt zu haben [12]. *ACTC1*-Mutationen wurden auch bei DCM gefunden, wobei sie dort weniger als 1 % der Fälle ausmachen [13]. Auch bei NDLVC und RCM wurde *ACTC1* beschrieben.

*DES* kodiert Desmin, ein Typ-3-Intermediärfilament, das Myofibrillen bündelt und im Sarkolemm verankert. Es spielt somit eine zentrale Rolle in der strukturellen und mechanischen Integrität des kontraktilen Apparats. Mutationen im *DES*-Gen werden zusammengefasst Desminopathien genannt und können zu kombinierten Krankheitsbildern von skelettalen Myopathien und CMP führen. RCM und ARVC sind mögliche Phänotypen einer *DES*-Mutation. *DES*-Mutationen sind wahrscheinlich in etwa 1 % der DCM-Fälle beteiligt, wurden aber ebenso bei HCM beschrieben.

Desmosomen sind Zellstrukturen in der Zellmembran, die Zell-Zell-Verbindungen herstellen und den mechanischen Zusammenhalt verbessern. Im Gegensatz zu den sarkomerischen Proteinen sind Mutationen in den 5 desmosomalen Genen *DSP* (Desmoplakin), *DSG2* (Desmoglein-2), *DSC2* (Desmocollin-2), *JUP* („junction plakoglobin“) und *PKP2* (Plakophilin-2) für bis zu 50 % aller Fälle der ARVC verantwortlich. Während *DSG2, DSC2, JUP* und *PKP2* nur bei ARVC beschrieben wurden, können Mutationen im *DSP*-Gen auch einen DCM-Phänotyp aufweisen. Patienten mit DCM und *DSP*-Mutationen weisen ein deutlich erhöhtes Risiko für maligne Arrhythmien auf [15].

Es gibt derzeit keine Therapie, die die zugrunde liegende genetische Veränderung korrigieren kann. Dennoch wird an einer Vielzahl von Methoden gearbeitet, die in Zukunft die genetischen Effekte modulieren könnten [16].

## Pädiatrische Patienten

Neu in der Leitlinie ist auch der Abschnitt, der sich mit dem Management von Kindern und Jugendlichen, die von CMP betroffen sind, befasst. Hauptsächlich handelt es sich hierbei um früh ausgeprägte Fälle mit entsprechend schlechter Prognose, aber auch um Fälle, bei denen eine milde phänotypische Expression familiärer CMP in der Regel durch Familienscreening erkannt wird. Die Leitlinie bietet erstmals Empfehlungen für das genetische Testen bei Kindern an, wobei der Zeitpunkt je nach Setting variabel sein kann. Zudem wird die Bedeutung des Übergangs von der pädiatrischen zur Erwachsenenversorgung betont.

## Generelles Management bei Kardiomyopathien

Im Abschnitt für das generelle Management werden die Strategien bezüglich Erhebung der Symptome, Therapie der Herzinsuffizienz sowie atrialer und ventrikulärer Arrhythmien, Device-Therapie und klinischer Kontrollen diskutiert.

Die Therapie der Herzinsuffizienz richtet sich nach der Leitlinie zur Herzinsuffizienz, wobei die dortigen Empfehlungen unabhängig von der Ätiologie gelten [17]. Die kardiale Amyloidose und die restriktive CMP benötigen jedoch ein spezielles Management, wobei die Euvolämie zentral ist [18]. Eine besondere Gruppe von Patienten sind diejenigen mit asymptomatischer linksventrikulärer Dysfunktion, die frühen Formen von CMP entsprechen kann. Da die Therapie der Herzinsuffizienz das Remodelling des Herzens positiv beeinflussen kann, sollte diese Therapie in Fällen von DCM oder NDLVC in Betracht gezogen werden (IIb C). Biomarker wie natriuretische Peptide könnten Risikopatienten identifizieren, die von einer frühen neurohumoralen Blockade profitieren könnten [19]. Bei Personen, die genetisch positiv, aber phänotypisch negativ sind, gibt es keine Hinweise auf den Nutzen einer pharmakologischen Therapie. Im Allgemeinen kann eine Herztransplantation (HTx) gemäß den üblichen Kriterien in Erwägung gezogen werden, insbesondere aber auch bei hochsymptomatischen Patienten mit HCM oder RCM, bei denen die linksventrikuläre Ejektionsfraktion (LVEF) normal ist, sowie bei Patienten mit therapierefraktären ventrikulären Arrhythmien (I C). Linksventrikuläre Herzunterstützungssysteme („left ventricular assist devices“, LVAD) können sowohl als „bridge-to-transplant“ (IIa B) als auch zur Verbesserung der Symptome und zur Reduzierung der Mortalität in Betracht gezogen werden (IIa B).

Bezüglich des Vorhofflimmerns ist bekannt, dass einige CMP wie die HCM und die kardiale Amyloidose mit einer höheren Schlaganfallrate assoziiert sind [20], sodass hier eine therapeutische Antikoagulation unabhängig vom CHA_2_DS_2_-VASc-Score eingeleitet werden sollte (IIa C; [21]). Eine Frequenzkontrolle sollte bei allen Patienten mit Vorhofflimmern und CMP erfolgen [22]. Betablocker sind die bevorzugte Wahl bei der pharmakologischen Therapie von CMP, während Digoxin als Alternative in Betracht gezogen werden kann. Verapamil oder Diltiazem sollten nur dann verwendet werden, wenn die LVEF über 40 % liegt. Bei unzureichender Frequenzkontrolle kann eine AV-nodale Ablation in Verbindung mit einer kardialen Resynchronisationstherapie (CRT) in Betracht gezogen werden. Bei der Rhythmuskontrolle sollte eine antiarrhythmische Therapie nur zurückhaltend verschrieben werden. Die Ablation ist eine sinnvolle Alternative, wobei die Rezidivrate von Vorhofflimmern bei CMP wahrscheinlich höher als normal liegt.

In Bezug auf ventrikuläre Arrhythmien gelten die ESC-Leitlinien von 2022 für das Management von ventrikulären Arrhythmien und die Prävention des SCD. Besonders bei der Implantation eines implantierbaren Kardioverter-Defibrillators (ICD) zur Primärprävention sollten einige spezielle Überlegungen berücksichtigt werden. Generell sollte das Arrhythmierisiko von Patienten mit CMP regelmäßig reevaluiert werden, idealerweise alle 1 bis 2 Jahre oder bei Veränderungen im klinischen Status (I C). Empfohlen werden Risikoscores/SCD-Algorithmus, die die Schwelle zur Implantation bei gleichzeitiger Betonung der partizipativen Entscheidungsfindung („shared decision making“) herabsetzen können.

### Klinische Kontrollen

Die Häufigkeit der klinischen Kontrollen sollte von der Schwere der Erkrankung, dem Alter und den Symptomen des Patienten abhängig gemacht werden. Im Allgemeinen sollte bei Patienten mit CMP alle 1 bis 2 Jahre eine klinische Untersuchung mit EKG und Echokardiographie durchgeführt werden (I C). Ebenso sollte eine Kontrolle erfolgen, wenn sich die Symptome unerwartet oder signifikant verschlechtern (I C). Die cMRT kann alle 2 bis 5 Jahre oder häufiger bei einer fortschreitenden Erkrankung in Betracht gezogen werden.

### Screening von Verwandten

**Figure 3:**
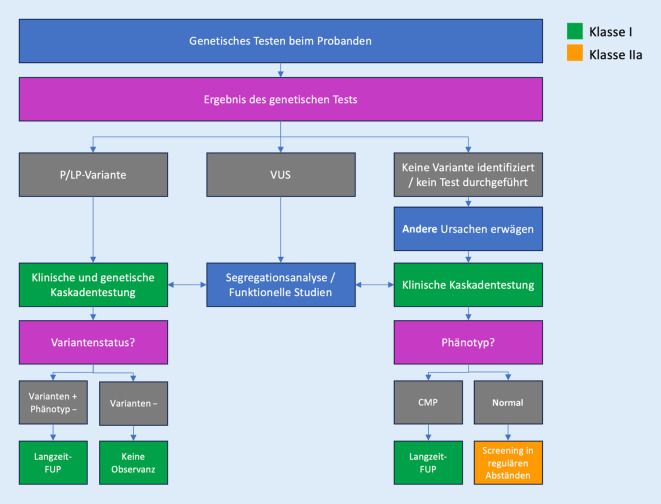

Neue Empfehlungen betreffen auch Verwandte von Patienten mit CMP und der gleichen genetischen Variante oder auch ohne Vorliegen eines genetischen Tests (Abb. 3).

Allen Verwandten 1. Grades eines Patienten mit einer CMP sollte eine kardiologische Untersuchung mit EKG und kardialer Bildgebung angeboten werden. In Familien, in denen eine pathogene oder wahrscheinlich pathogene („pathogenic/likely pathogenic“, P/LP) Variante festgestellt wurde, sollte eine Kaskadentestung angeboten werden. Individuen, die keinen klinischen Phänotyp aufweisen und bei denen keine P/LP-Variante identifiziert wurde, können von einer engmaschigen Überwachung befreit werden (I C). Bei Vorliegen der gleichen Variante werden regelmäßige klinische Untersuchungen empfohlen, wobei die Häufigkeit vom Alter, vom Schweregrad der Erkrankung in der Familie und vom Genotyp abhängt. Wenn beim Patienten keine P/LP-Variante festgestellt wurde oder wenn keine genetischen Tests durchgeführt wurden, sollten alle Verwandten 1. Grades klinisch nachverfolgt werden (I C). In Familien, in denen keine P/LP-Variante festgestellt wurde, sollte trotzdem eine klinische Beobachtung für Kinder angeboten werden. In Familien, in denen nur 1 Person eine CMP hat und keine genetische Variante identifiziert wurde, kann die Häufigkeit der klinischen Kontrollen bei Verwandten reduziert werden. Bei Risikopatienten, die Träger einer P/LP-Variante sind oder bei denen eine familiäre CMP vorliegt, sollte das Screening bis zum 60. Lebensjahr alle 1 bis 3 Jahre und danach alle 3 bis 5 Jahre erfolgen.

## Management der verschiedenen Phänotypen

### Hypertrophe Kardiomyopathie

Die Empfehlungen für die HCM wurden anhand der letzten ESC-Leitlinie von 2014 aktualisiert. In Bezug auf die pharmakologische Therapie der HCM mit linksventrikulärer Ausflusstraktobstruktion (LVOTO) wurde Mavacamten, ein kardialer First-in-class-Myosin-ATPase-Inhibitor, aufgrund der Ergebnisse der Studien EXPLORER-HCM und VALOR-HCM in die Leitlinie aufgenommen [23–25]. Mavacamten kann als Second-line-Therapie in Betracht gezogen werden und mit Betablockern und Kalziumantagonisten, aber nicht mit Disopyramid, kombiniert werden (IIa A). Eine Monotherapie bei Unverträglichkeit oder Kontraindikation für andere Substanzen ist auch möglich (IIa B). Die Dosissteigerung sollte unter echokardiographischer Überwachung erfolgen.

**Figure 4:**
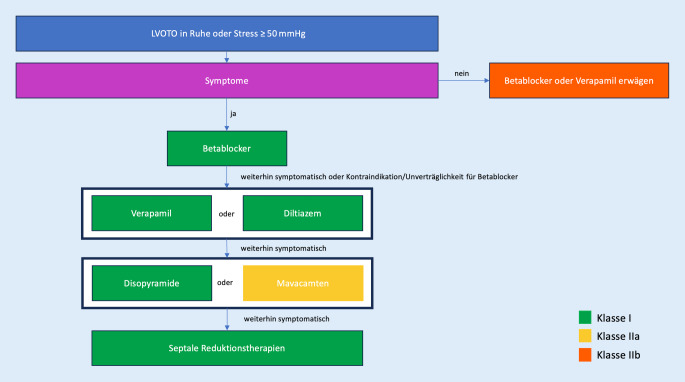

Die Therapie bei LVOTO ist in Abb. 4 dargestellt. In Bezug auf die Indikation für eine CRT betont die Leitlinie, dass bei HCM eher die alleinigen EKG-Kriterien für eine CRT herangezogen werden sollten, ohne dass zusätzlich eine signifikante Reduzierung der LVEF vorliegen muss, was bei HCM seltener vorkommt. Da es jedoch keine ausreichende Datenlage gibt, verzichtet die Leitlinie hier auf spezifische Empfehlungen.

Ein weiterer wichtiger Abschnitt betrifft die Prävention des SCD. Hier empfiehlt die Leitlinie eine Risikoevaluierung mittels Risikorechnern (https://qxmd.com/calculate/calculator_303/hcm-risk-scd) als ersten Schritt [26–28]. Bei einem 5‑Jahres-Risiko von mehr als 6 % sollte eine primärprophylaktische ICD-Implantation erwogen werden (IIa B), bei 4–6 % kann sie in Betracht gezogen werden, und bei weniger als 4 % können ein hohes LGE („late gadolinium enhancement“; 15 %) oder eine LVEF unter 50 % als zusätzliche Risikofaktoren bei der Entscheidungsfindung mitberücksichtigt werden (IIb B).

### Dilatative Kardiomyopathie

Für das Management der DCM gelten die Leitlinien für die Herzinsuffizienz und die implantierbaren Geräte (Devices) entsprechend den Empfehlungen [17, 29]. Empfehlungen für eine asymptomatische linksventrikuläre Dysfunktion oder eine linksventrikuläre Dilatation sind selten, was eine Herausforderung bei genetisch bedingter DCM darstellen kann, da viele asymptomatische Patienten in jungem Alter durch das Screening identifiziert werden. Neue Forschungsergebnisse zeigen auch, dass der genetische Hintergrund eine Rolle beim Auftreten von Arrhythmien spielt. Bestimmte genetische Mutationen erhöhen das Arrhythmierisiko unabhängig von der LVEF [30–35]. Bei diesen Patienten könnte die primärprophylaktische Implantation eines ICD auch bei einer LVEF von mehr als 35 % in Erwägung gezogen werden, insbesondere dann, wenn andere Risikofaktoren wie nichtanhaltende ventrikuläre Tachykardien („non-sustained ventricular tachycardias“, nsVT), ventrikuläre Ektopie oder ausgeprägtes LGE in der cMRT vorhanden sind. Zurzeit laufen Studien, die die Bedeutung von LGE bei der DCM im Zusammenhang mit dem SCD genauer untersuchen (NCT04558723 und NCT03993730). Für manche Gene oder bestimmte Varianten gibt es Risikoscores, wie z. B. für die LMNA-CMP (https://lmna-risk-vta.fr; [36]). Die Empfehlungen für die Prävention des SCD und die Auflistung der Risikogene zeigt Tab. 4.

**Table 4:** 

	Empfehlungsgrad	Evidenz
*Sekundärprävention*		
Eine ICD-Implantation wird für Patienten mit einer DCM/NDLVC, die einen SCD oder ventrikuläre Arhythmien mit hämodynamischer Instabilität überlebt haben, empfohlen, um Mortalität und SCD zu senken	I	B/C
*Primärprävention*		
Eine ICD-Implantation sollte für Patienten mit einer DCM/NDLVC und symptomatischer Herzinsuffizienz sowie LVEF ≤ 35 % trotz OMT > 3 Monate in Betracht gezogen werden	IIa	A
Der Genotyp sollte bei der Risikoabschätzung des SCD bei DCM/NDLVC berücksichtigt werden	IIa	B/C
Eine ICD-Implantation sollte für Patienten mit einer DCM/NDLVC und einem Hochrisikogenotyp auch bei einer LVEF > 35 % und zusätzlichen Risikofaktoren erwogen werden	IIa	C
Eine ICD-Implantation kann bei ausgewählten Patienten mit einer DCM/NDLVC und einem Hochrisikogenotyp auch bei einer LVEF > 35 % ohne zusätzliche Risikofaktoren erwogen werden	IIb	C
Eine ICD-Implantation kann bei Patienten mit einer DCM/NDLVC ohne einen Hochrisikogenotyp auch bei einer LVEF > 35 % und zusätzlichen Risikofaktoren erwogen werden	IIb	C

Hochrisikogene: *LMNA* (Lamin A/C), *FLCN* (Folliculin; „truncating variants“), *TMEM43* („transmembrane protein 43“), *PLN* (Phospholamban), *DSP* (Desmoplakin), *RBM20* („RNA-binding motif protein 20“)

Zusätzliche Risikofaktoren sind unter anderem eine Synkope oder substanzielles LGE („late gadolinium enhancement“) in der kardialen Magnetresonanztomographie (cMRT)

*CMP* Kardiomyopathie, *NDLVC* nichtdilatierte linksventrikuläre CMP, *SCD* „sudden cardiac death“, *OMT* „optimal medical therapy“, *LVEF* linksventrikuläre Ejektionsfraktion

### Nichtdilatierte linksventrikuläre Kardiomyopathie

Patienten mit dem NDLVC-Phänotyp sollten einem diagnostischen Work-up unterzogen werden, wie in den Einführungsabschnitten beschrieben. NDLVC sollte als familiär betrachtet werden, wenn ein oder mehrere Verwandte 1. oder 2. Grades ebenfalls NDLVC haben oder wenn ein Verwandter 1. Grades einen SCD aufgrund von NDLVC erlitten hat. Die am häufigsten betroffenen Gene sind *DSP, FLCN* (Folliculin), *DES, LMNA* und *PLN* (Phospholamban). Die Empfehlungen für eine ICD-Implantation entsprechen weitgehend denen für die DCM, mit nur geringfügigen Unterschieden in einigen Evidenzgraden (Tab. 4).

### Arrhythmogene rechtsventrikuläre Kardiomyopathie

Die ARVC manifestiert sich am ehesten zwischen der 2. und 4. Lebensdekade und betrifft häufiger Männer. Es wurden eine altersabhängige Penetranz sowie eine hohe klinische und genetische Variabilität beschrieben. Eine ARVC sollte bei jungen Erwachsenen mit Palpitationen, Synkopen oder einem überlebten SCD in Betracht gezogen werden. Die ARVC präsentiert sich oft mit Linksschenkelblock (LSB), ventrikulären extrasystolischen Schlägen (VES) oder VT, manchmal begleitet von einer rechtsventrikulären Dilatation. Die cMRT ist die einzige Untersuchung, die die Beteiligung des linken Ventrikels durch Darstellung der fettigen Degeneraton abschätzen kann. Betablocker sind die erste Wahl zur Kontrolle von VES, nsVT und VT (I C). Obwohl die Erkrankung selten ist, wird die ARVC in Registern als eine häufige Ursache für einen SCD erkannt [37]. Der überarbeitete 2019er ARVC-Risiko-Rechner sollte zum Abschätzen des Risikos herangezogen werden (IIa B), auch Hochrisikomerkmalen sollte eine Bedeutung beigemessen werden (IIa B; [38–41]).

### Restriktive Kardiomyopathie

Die RCM gehört zu den CMP mit der schlechtesten Prognose. Mehr als 75 % der überlebenden Patienten präsentieren sich mit Herzinsuffizienz, und ohne Herztransplantation führt die Erkrankung binnen weniger Jahre zum Tod [42–44]. Die pulmonalen Drücke können rasch, auch ohne klinische Veränderungen, ansteigen, was bei der zeitlichen Planung einer Herztransplantation berücksichtigt werden muss.

## Weitere Abschnitte

In den weiteren Abschnitten behandelt die Leitlinie die syndromischen CMP und gibt Empfehlungen bezüglich körperlicher Aktivität und Training, Management in der Schwangerschaft oder nichtkardialer Operationen und Lebensführung.

Insgesamt ist die neue Leitlinie eine notwendige Ergänzung der bisherigen Leitlinien für Herzinsuffizienz und ist von großer Bedeutung für die klinische Praxis und die Patientenversorgung. Sie betont die Wichtigkeit der Ursachensuche bei CMP unklarer Genese und legt einen starken Fokus auf eine genetische Beratung. Je nach zugrunde liegender Pathologie werden unterschiedliche Behandlungen empfohlen, was eine individualisierte Herangehensweise und gleichzeitig eine ganzheitliche Betreuung fördert. Eine Besonderheit besteht darin, dass Empfehlungen nicht nur für Patienten mit CMP, sondern auch für deren Familienmitglieder gegeben werden, insbesondere für Personen mit erhöhtem Risiko.
